# Comparison of fentanyl versus meperidine as supplements to epidural clonidine-bupivacaine in patients with lower limb orthopedic surgery under combined spinal epidural anesthesia

**DOI:** 10.1186/s12871-015-0126-5

**Published:** 2015-10-14

**Authors:** Ayman Abd Al-maksoud Yousef, Ashraf Mohamed Atef, Waleed Mohamed Awais

**Affiliations:** 1 Anesthesia Department, Faculty of Medicine, Tanta University, Alguish street, Tanta, Egypt; 2 Department of Orthopedic Surgery, Faculty of Medicine, Tanta University, Tanta, Egypt

## Abstract

**Background:**

The analgesic and sedative effect of clonidine explain its common use as adjuvant in regional anesthesia, however the hemodynamic instability associated with its neuroaxial administration is the major drawback. Our study hypothesis is to compare the hemodynamic and analgesic effect of epidural fentanyl in comparison to meperidine when added to clonidine in patients undergoing lower limbs orthopedic surgery using combined spinal–epidural anesthesia.

**Methods:**

One hundred thirty five ASA physical status I or II patients were recruited for lower limb orthopedic surgery. All received 2 mL intrathecal 0.5 % hyperbaric bupivacaine, 10 mL epidural 0.25 % plain bupivacaine, and 1 mL epidural clonidine 2 μg/kg (Clonidine group) and then either 1 ml fentanyl 25 μg (Fentanyl Group) or 1 ml meperidine 25 mg (Meperidine Group). The quality of surgical anesthesia, incidence of hypotension and bradycardia, intra-operative pain assessment, and onset of postoperative pain, sedation scores and side effects in the postoperative period were recorded.

**Results:**

The 1^st^ analgesic requirement in the postoperative period was significantly prolonged in the meperidine group (*p* = 0.001). Significant decrease in the mean arterial blood pressure in fentanyl group was at 15, 30, 45, 60 and 90 min (*p* = 0.035, 0.019, 0.027, 0.032 and 0.039) respectively, significant decrease in meperdine group was at 15 and 30 min (*p* = 0.038 and 0.043), while in clonidine group a significant decrease was at 15, 30, 45, and 60 min (*p* = 0.025, 0.028, 0.036 and 0.042) respectively. Among group changes, the mean arterial blood pressure was significantly higher in meperdine group at 30, 45, 60 and 90 min (*p* = 0.007, 0.015, 0.029 and 0.033) respectively. A significant decrease in the heart rate in fentanyl group at 15, 30 and 45 min (*p* = 0.035, 0.018 and 0.029), in meperdine group a significant decrease in the heart rate was at 15 min (*p* = 0.038), while in clonidine group a significant decrease was at 15, and 30 min (*p* = 0.016 and 0.003) . Among group changes, the heart rate was significantly higher in meperdine group at 30, 45 and 60 min (*p* = 0.021, 0.017 and 0.011). VAS were significantly lower in meperdine group in comparison to fentanyl and clonidine groups at 2 h, 3 h and 4 h post-operative period (*p* = 0.024, 0.001 and 0.039).

**Conclusion:**

The combined administration of epidural clonidine and meperidine provided better intraoperative hemodynamics and prolonged postoperative analgesia than epidural clonidine fentanyl combination in patients undergoing lower limb orthopedic surgery.

**Trial registration:**

Clinical Trail Registry (Clinicaltrail.gov) NCT02128451.

## Background

Regional anesthesia is preferred choice for surgery of the lower limbs; the use of epidural local anesthetics alone is inconsistent in maintaining a level of sensory analgesia in the postoperative period [1].

The combined use of local anesthetics with adjuvant drugs is aimed to improve the quality of intra-operative anesthesia, postoperative analgesia, aid early ambulation, early recovery of motor block, reducing the incidence of associated side effects; is the concept behind the multimodal analgesia [2].

Opioids and *α*- 2adrenergic agonists interact synergistically rather than additively to produce analgesia for postoperative pain relief, thus combination therapy, by reducing the dose of each, could reduce the incidence and severity of their side effects [3].

The optimum opioid for epidural analgesia is still controversial; however nausea pruritus and respiratory depression are the main side effects of opioid use. Meperidine has intermediate lipophilicity, provides postoperative analgesia of good quality, of prolonged duration and not associated with late respiratory depression. Thus, meperidine may be a preferred choice of epidural opioid [4].

Therefore, our study hypothesis is to compare the hemodynamic and the analgesic properties of epidural meperidine versus fentanyl when added to epidural clonidine in patients undergoing lower limbs orthopedic surgery using combined spinal–epidural anesthesia.

## Methods

The study was approved by an Investigational Review Board of Faculty of Medicine, Tanta University; an informed written consent was obtained from all patients participating in the study. This study was registered in Clinical Trail Registry (Clinicaltrail.gov) with unique identification number NCT02128451.

One hundred thirty five healthy (ASA I, II), 18–60 year old patients admitted to Orthopedic Department, Faculty of Medicine, Tanta University for lower limb surgery were allocated. Randomization was performed by random numbers using sealed envelopes without sex stratification. Sealed envelopes indicate the group of assignment. Patients were blindly randomized to the three groups, 45 in each group, the process of inclusion into the study went on until the requested number of patients was reached.

Patients with pre-existing hypertension requiring treatment, hepatorenal or other end organ disease, patients received opioid agonist or agonist/antagonist in the preceding 6 h (or within 1 h if given intravenously), patients with morbid obesity (BMI >40 kg/m^**2**^), patients with extremes contraindications of height (<140 or >180 cm) were excluded from the study, patients with coagulation or neurological disorders, spine deformity, or skin infection were also excluded.

In the operating theatre patients were continuously monitored using electrocardiogram, pulse oximeter, while non- invasive blood pressure was measured every 5 min for the first 15 min and then every 15 min throughout the intra-operative period. In the sitting position, CSE anesthesia was performed using a needle-through-needle technique at L3–4 interspace using a midline approach (Epi-Star CSE, Maxi-Set, Kemen, Germany). An independent anesthesiologist, who did not participate in the study or data collection, prepared unlabeled syringes containing the study drugs. The epidural space is located using loss of resistance to air with an 18-gauge Tuohy needle, and dural puncture was achieved with 27-gauge pencil point needle. After confirmatory aspiration of cerebrospinal fluid, 2 mL of 0.5 % hyperbaric bupivacaine was injected intrathecally. The spinal needle was withdrawn, and epidural injection was started in the form of 12 mL of the study solution. This was 10 mL 0.25 % plain bupivacaine, 1 ml clonidine 2 μg/kg plus, 1 ml normal saline (Clonidine group) 1 ml fentanyl 25 μg (Fentanyl Group) or 1 ml meperidine 25 mg (Meperidine Group).

The level of sensory block was assessed at 2 min intervals for 30 min after epidural injection using pinprick. The highest level of sensory block (S max) and time taken to reach S max were recorded. The time needed for the block to reach the dermatome of the 10th thoracic segment which is the level of readiness for surgery, and time interval between anesthesia and recovery from motor blockade were recorded. The time to 1^st^ analgesic requirement in the postoperative period was also recorded.

Motor blockade of the lower extremities was assessed at 5 min intervals for 30 min using the modified Bromage score (BS): BS0, full flexion of hip, knee and ankle; BS1, impaired hip flexion; BS2, impaired hip and knee flexion; BS3, unable to flex hip, knee or ankle. Complete motor block was defined as BS3.

Sedation was assessed using Subjective Sedation Scale (Grade 0: fully awake, Grade 1: calm, Grade 2: awake on verbal command, Grade 3: awake on gentle tactile stimulation, Grade 4: awake on vigorous stimulation and Grade 5: unarousable).

Hypotension is the fall in the mean arterial blood pressure 20 % from pre-induction levels or a systolic blood pressure lower than 100 mmHg, and treated immediately with 5 mg ephedrine by intravenous injection. Bradycardia is the decrement of heart rate bellow 50 beats/min and treated immediately with 0.5 mg atropine by intravenous injection.

Intra-operative and post-operative pain were assessed on a 10-cm visual analogue scale (VAS), in which 0 represented no pain and 10 represented worst possible pain. VAS was measured every 15 min intra-operatively and every hour post-operatively for the first 4 h, then every 4 h for the 1^st^ 24 postoperative hours by an anesthesiologist who was unaware of the patient allocation group. All patients received 15 mg/kg i. v. acetaminophen every 6 h; the first dose was given before completion of surgery. If patient complained of pain (defined as VAS >4), intravenous rescue fentanyl was given in 50 μg increments**.** Patients with VAS <4 were given their acetaminophen infusion [5–9].

The requirement for supplementary analgesia was noted in different groups. Adverse effects such as hypotension, bradycardia, nausea, vomiting, pruritus, and shivering were also recorded.

Surgery was performed by one of two consultant surgeons of similar clinical experience; they were blinded to the allocation group. Following surgery, patients were nursed in the PACU. Recovery from motor block was defined as time from injection of epidural solution to BS0. The onset of postoperative pain, defined as the time from completion of surgery to onset of VAS >4, was recorded. The primary outcome measure was the changes in the incidence of hypotension. Secondary end points included the assessment of quality of intraoperative anesthesia, sedation score and duration of postoperative analgesia.

### Statistical analysis

Continuous data were presented as mean (±SD). Parametric data were analyzed using Student’s *t* test while, non-parametric data were analyzed using Mann–Whitney *U*, and categorical data were assessed with _***X***_^***2***^ tests. A *P* value <0.05 was considered significant.

### Sample size analysis

A sample size analysis using data obtained in an initial pilot study indicated that 45 patients per group were required to detect a between group difference of at least 20 % in the incidence of hypotension, with a power of 80 %, α of 0.05.

## Results

A total of 193 patients were considered for entry into the study. Of these 28 did not meet the inclusion criteria and 30 refused to participate. The remaining 135 patients were randomized into three study groups. The three groups had similar characteristics with regard to age, sex, height, weight, type and duration of surgery (*p* = 0.695, 0.789, 0.352, 0.071, 0.983 and 0.541) respectively (Table 1).

**Table 1:** Patient characteristics in the studied groups

	Fentanyl group (*n* = 45)	Meperidine group (*n* = 45)	Clonidine group (*n* = 45)	Analysis of variance Or *X*^2^	*P*-value
Age (years)	48.9 ± 17.4	45.6 ± 14.9	43.5 ± 13.5	0.366	0.695
Sex (male/female)	29/16	32/13	30/15	0.472	0.789
Height (cm)	174.5 ± 12.4	169.6 ± 13.5	171.4 ± 12.7	0.745	0.352
Weight (kg)	93.6 ± 14.6	89.7 ± 15.4	90.5 ± 12.2	0.815	0.071
Type of surgery: Ankle fracture	19	16	18		
	15	14	13	0.3813	0.983
		15	14		
Tabial fracture Arthroscopy					
Duration of surgery (min)	115.7	109.5	118.7	0.76	0.541

Data are mean ± standard deviation or number (%)

There were no statistically significant difference between groups regarding highest level of sensory block (S max) (*p* = 0.968), the time needed for the block to reach the S max (*p* = 0.495) and the dermatome of the 10th thoracic segment (*p* = 0.675), the duration of motor (*p* = 0.074) and the grade of motor blockade (*p* = 0.879). The time to 1^st^ analgesic requirement in the postoperative period was significantly prolonged in the meperidine group (*p* = 0.001) (Table 2).

**Table 2:** Block characteristics in the studied groups

Block characteristics	Fentanyl group	Meperidine group	Clonidine group	Analysis of variance or *X*^2^	
				F or *X*^2^	*P*
	(*n* = 45)	(*n* = 45)	(*n* = 45)		
Highest level of sensory blockade. T9	17 (37.7 %)	20 (44.4 %)	19 (42.2 %)	1.36	0.968
T8	12 (26.6 %)	14 (31.1 %)	13 (28.8 %)		
T7	10 (22.2 %)	7 (15.5 %)	8 (17.7 %)		
T6	6 (13.3 %)	4 (8.8 %)	5 (11.1 %)		
Time to reach sensory block. (min)	5.4 ± 1.4	5.8 ± 1.2	5.5 ± 1.1	0.829	0.652
Time to S max. (min)	7.5 ± 1.5	8.1 ± 1.7	7.8 ± 1.4	0.755	0.495
Time to ready for surgery. (min)	6.4 ± 1.3	7.5 ± 1.6	6.9 ± 1.5	0.582	0.675
Duration of motor block. (min)	148 ± 36	133.5 ± 40	140 ± 35	4.95	0.074
Grade of motor blockade	10	9	11	0.257	0.879
BS 2	35	36	34		
BS 3					
Time to 1^st^ analgesic requirement. (min)	197 ± 56	318 ± 72	155 ± 42	150.48	0.001

Data are mean ± standard deviation or number (%)

BS: Bromage scale

There is no significant difference between the groups regarding the mean value of mean arterial blood pressure in the pre-operative period. Regarding the arterial blood pressure within each group in comparison to the pre-operative mean value, there was a significant decrease in the mean arterial blood pressure in fentanyl group at 15, 30, 45, 60 and 90 min (*p* = 0.035, 0.019, 0.027, 0.032 and 0.039) respectively, in meperdine group there was a significant decrease in the mean arterial blood pressure at 15 and 30 min (*p* = 0.038 and 0.043) respectively, while in clonidine group there was a significant decrease in the mean arterial blood pressure at 15, 30, 45, and 60 min (*p* = 0.025, 0.028, 0.036 and 0.042) respectively. Among group changes, the mean arterial blood pressure was significantly higher in meperdine group at 30, 45, 60 and 90 min (*p* = 0.007, 0.015, 0.029 and 0.033) in comparison to other groups respectively (Table 3).

**Table 3:** Comparison of mean values of mean arterial blood pressure in the studied groups

	Pre-ope	5 min	15 min	30 min	45 min	60 min	90 min	120 min	3 h	6 h	12 h	24 h
Fentanyl group (*n* = 45)	90.6 ± 17.8	86.7 ± 14.5	81.5^a^ ± 12.9	70.5^a^ ± 13.8	75.6^a^ ± 12.5	77.8^a^ ± 13.6	78.5^a^ ± 12.8	81.2 ± 12.5	85.5 ± 13.9	88.2 ± 14.5	89.7 ± 16.8	91.3 ± 17.5
Meperidine group (*n* = 45)	89.7 ± 14.9	85.9 ± 16.3	79.2^a^ ± 12.3	80.5^ab^ ± 18.5	82.9^b^ ± 13.1	83.3^b^12.4	84.5^b^ ± 14.5	85.8± 12.1	86.4 ± 15.3	87.3 ± 14.7	88.5 ± 15.3	90.2 ± 12.5
Clonidine group (*n* = 45)	92.5 ± 19.5	86.5 ± 17.2	80.4^a^ ± 16.9	72.2^a^ ± 15.4	76.4^a^ ± 17.5	78.5^a^ ± 16.8	82.8 ± 14.7	84.5 ± 13.5	86.7 ± 16.3	88.4 ± 15.7	90.2 ± 14.1	91.5 ± 17.5

^a^Significant within group change (*p* < 0.05)

^b^Significant among group change (*p* < 0.05)

Data are mean ± standard deviation

There is no significant difference between the groups regarding the mean value of mean heart rate in the pre-operative period (*p* = 0.572). Regarding the mean value of heart rate within each group in comparison to the pre-operative value, there was a significant decrease in the heart rate in fentanyl group at 15, 30 and 45 min (*p* = 0.035, 0.018 and 0.029) respectively, in meperdine group there was a significant decrease in the heart rate at 15 min (*p* = 0.038), while in clonidine group there was a significant decrease in heart rate at 15, and 30 min (*p* = 0.016 and 0.003) respectively. Among group changes, the heart rate was significantly higher in meperdine group at 30, 45 and 60 min (*p* = 0.021, 0.017 and 0.011) in comparison to other groups respectively (Table 4).

**Table 4:** Comparison of mean values of heart rate in the studied groups

	Pre-ope	5 min	15 min	30 min	45 min	60 min	90 min	120 min	3 h	6 h	12 h	24 h
Fentanyl group (*n* = 45)	87.7 ± 14.9	82.6 ± 13.7	77.5^a^ ± 14.5	72.9^a^ ± 12.5	75.5^a^ ± 16.4	78.5 ± 15.5	81.6 ± 11.7	82.3 ± 13.6	83.6 ± 15.4	84.3 ± 16.7	84.8 ± 18.3	85.4 ± 17.8
Meperidine group (*n* = 45)	86.8 ± 15.5	81.3 ± 13.4	76.2^a^ ± 12.84	81.3^b^ ± 18.5	83.4^b^ ± 13.6	83.7^b^ ± 15.8	84.9 ± 12.7	85.4 ± 14.3	86.5 ± 16.9	87.2 ± 14.7	87.8 ± 11.5	88.6 ± 16.4
Clonidine group (*n* = 45)	85.5 ± 19.2	85.7 ± 15.8	78.2^a^ ± 16.9	74.4^a^ ± 17.3	76.5 ± 16.l	78.7 ± 15.7	82.9 ± 18.5	83.2 ± 14.8	84.5 ± 12.6	85.7 ± 13.5	86.3 ± 11.9	87.6 ± 12.8

^a^Significant within group change (*p* <0.05)

^b^Significant among groups change (*p* <0.05)

Data are mean ± standard deviation

Regarding the VAS in studied groups, there is no significant changes among groups in the immediate postoperative period (*p* = 0.655). In meperdine group, the VAS were significantly lower in comparison to mean values of fentanyl and clonidine groups mean values at 2 h, 3 h and 4 h post-operative period (*p* = 0.024, 0.001 and 0.039) (Table 5).

**Table 5:** Comparison of mean values of VAS in the studied groups

VAS	Fentanyl group (*n* = 45)	Meperidine group (*n* = 45)	Clonidine group (*n* = 45)
Immediate post-operative	0.95 ± 0.35	0.75 ± 0.29	1.1 ± 0.42
1- h	1.2 ± 0.46	0.87 ± 0.39	1.23 ± 0.42
2- h	1.69 ± 0.52	1.17^b^ ± 0.29	2.95^a^ ± 0.85
3- h	3.27^a^ ± 1.15	1.34^b^ ± .28	3.82^a^ ± 1.35
4- h	3.72^a^ ± 0.88	2.75^ab^ ± 0.95	4.23^a^ ± 0.74
8- h	4.15^a^ ± 1.52	3.98^a^ ± 1.38	4.55^a^ ± 1.33
12- h	4.37^a^ ± 1.4	4.15^a^ ± 1.26	4.66^a^ ± 1.19
16- h	4.52^a^ ± 1.29	4.36^a^ ± 1.49	4.65^a^ ± 1.37
24- h	4.65^a^ ± 1.35	4.45^a^ ± 1.22	4.72^a^ ± 1.16

^a^Significant within group change (*p* <0.05), ^b^Significant among groups change (*p* <0.05)

Data are mean ± standard deviation

There was no statistically significant difference regarding the different grades of the sedation score in studied groups (*p* = 0.986) (Table 6).

**Table 6:** Intra-operative sedation score in the studied groups

Intra-operative sedation score	Fentanyl group (*n* = 45)	Meperidine group (*n* = 45)	Clonidine group (*n* = 45)	Chi-square	
				*X* ^2^	*P*-value
Grade 0	4	5	5	0.981	0.986
Grade 1	19	17	18		
Grade 2	14	15	12		
Grade 3	7	8	5		

Data are number (%)

None of the patients required intraoperative analgesic supplementation in studied groups. Significantly higher number of patients required supplementary postoperative analgesia in fentanyl and clonidine groups in comparison to meperidine group (p = 0.0009). The mean postoperative fentanyl requirement was significantly reduced in meperidine group (*p* = 0.027) (Table 7).

**Table 7:** Supplementary analgesia in the studied groups

Supplementary fentanyl	Fentanyl group (*n* = 45)	Meperidine group (*n* = 45)	Clonidine group (*n* = 45)	Chi-square or analysis of variance	
				*X*^2^ and F	*P*-value
Post-operative requirement. number (%)	17(37.7 %)	6(13.3 %)	21(46 %)	*X*^2^ = 13.9	0.0009
Mean total fentanyl usage (μg)	18.8	6.6	23.3	F = 9.54	0.027

Data are mean ± standard deviation or number (%)

There were statistically significant lower numbers of patients in meperdine group suffered from intraoperative hypotension and bradycardia in comparison to fentanyl and clonidine groups (*p* = 0.011 and 0.009) respectively. There were statistically significant higher numbers of patients in fentanyl group suffered post-operative shivering and required oxygen mask for its control (*p* = 0.002). There were statistically significant higher numbers of patients suffered from itching in meperidine group. (*P* = 0.029) There were no statistically significant difference between groups regarding the incidence of nausea, vomiting (Table 8).

**Table 8:** Incidence of side effects in both groups

	Fentanyl group (*n* = 45)	Meperidine group (*n* = 45)	Clonidine group (*n* = 45)	Chi-square	
				*X* ^2^	*P*-value
Hypotension	16	4	11	8.86	0.011
Bradycardia	13	2	8	9.34	0.009
Nausea and vomiting	4	3	3	0.214	0.898
Itching	3	8	1	7.06	0.029
Shivering	10	2	1	11.65	0.002

Data are number (%)

## Discussion

The results of this study demonstrated that epidural meperidine 25 mg, in addition to a standardized CSE dosage regimen using epidural clonidine and bupivacaine achieved better intra-operative hemodynamics without affecting the degree of sedation beside prolonged postoperative analgesia as the VAS were significantly lower at post-operative period. The use of such combination of clonidine and meperidine in these doses avoids the occurrence of hemodynamic instability such as hypotension and bradycardia in addition, to prolonged postoperative analgesia.

Fentanyl is highly lipophilic; it undergoes rapid vascular absorption from the epidural space, it undergoes less rostral spread leading to relatively rapid analgesic of relatively short duration with limited dermatomal spread [10, 11].

Unlike fentanyl, meperidine is an opioid of intermediate lipophilicity, has local anesthetic activity, when injected into the epidural space it is absorbed into CSF rapidly with maximal levels after 15 min, thus providing analgesia that is of early onset but of relatively prolonged duration without hemodynamic deterioration [12]. Meperidine has selective spinal action at the dorsal horn cells change, into. In addition, meperdine has sympathomimetic activity upon systemic absorption producing increase in cardiac output and heart rate [13].

Previous studies [14–16] compared the analgesic efficacy of epidural meperdine and fentanyl with nearly similar finding to the present study, Paech, [14] compared the epidural meperdine or fentanyl during caesarean section and concluded that the quality of analgesia and duration of effective analgesia was good in both groups with minimum side effects. Cox et al., [15] compared epidural fentanyl and meperdine with bupivacaine in the management of postoperative pain after major abdominal surgery. Postoperative pain scores using a visual analogue scale were not significantly different between the two groups. Goh et al., [16] compared fentanyl and meperdine for postoperative epidural analgesia in women having elective caesarean deliveries and conclude that meperdine is a suitable drug for patient-controlled epidural analgesia with greater patient satisfaction when compared to fentanyl.

Similar to our experience multiple clinical studies were performed evaluating the value of epidural meperdine and demonstrated comparable effects to this study; Baraka et al., [17] demonstrated that epidural meperidine was associated with stable hemodynamics, satisfactory analgesia and reduced the need for administration of rescue analgesics when administered epidurally for obstetric patients. Cardiac output and mean arterial pressure was increased after epidural injection of meperidine [15].

In addition; St-Onge et al., [13] in their study demonstrated that bupivacaine decreases epidural meperidine requirements after abdominal surgery producing satisfactory analgesia without increasing the incidence of side effects. Ferrante et al., [1] in their study in patients undergoing total knee replacement concluded that patients received epidural bupivacaine meperidine combination had a significantly slower regression of sensory anesthesia and slower development of postoperative pain in comparison to patients receiving either bupivacaine alone or bupivacaine with fentanyl. Robinson et al., [18] in their study regarding the analgesic effect of epidural meperidine after cardiac surgery confirmed the relatively prolonged analgesia produced by meperedine in comparison to other opioids without affecting the degree of somnolence.

## Conclusion

The combined administration of epidural clonidine and meperdine provided better intraoperative hemodynamics and prolonged postoperative analgesia than epidural clonidine fentanyl combination in patients undergoing lower limb orthopedic surgery.

## Abbreviations

ASA: American society of anesthesiologist
BMI: body mass index
PACU: post-anesthesia care unit
CSE: combined spinal epidural
